# Advances in understanding the role of immune checkpoint LAG-3 in tumor immunity: a comprehensive review

**DOI:** 10.3389/fonc.2024.1402837

**Published:** 2024-08-26

**Authors:** Yingzhe Luo, Xuebin Cai, Biao Yang, Facheng Lu, Cheng Yi, Guoyu Wu

**Affiliations:** ^1^ Department of Oncology, Hospital of Chengdu University of Traditional Chinese Medicine, Chengdu, Sichuan, China; ^2^ Department of Abdominal Oncology, Division of Medical Oncology, Cancer Center, West China Hospital, Sichuan University, Chengdu, Sichuan, China

**Keywords:** LAG-3, immunotherapy, relatlimab, tumor, PD-1

## Abstract

Lymphocyte activation gene 3 (LAG-3), also known as CD223, is an emerging immune checkpoint that follows PD-1 and CTLA-4. Several LAG-3 targeting inhibitors in clinical trials and the combination of relatlimab (anti-LAG-3) and nivolumab (anti-PD-1) have been approved for treating - unresectable or metastatic melanoma. Despite the encouraging clinical potential of LAG-3, the physiological function and mechanism of action in tumors are still not well understood. In this review, we systematically summarized the structure of LAG-3, ligands of LAG-3, cell-specific functions and signaling of LAG-3, and the current status of LAG-3 inhibitors under development.

## 1 Introduction

Using the mechanism of immune checkpoint and tumor cells to implement immunotherapy or develop antibodies is a promising direction of antitumor therapy. PD-1 and CTLA-4, the two most classic immune checkpoints of tumor immunotherapy, have problems with immune tolerance and limited response rate while inducing long-lasting anti-tumor response (1–3). LAG-3, identified in 1990 as a CD4 structural homolog, is expressed by a diversity of lymphocytic and nonlymphocytic lineage cells (4). Recent studies have identified that LAG-3, along with PD-1 and CTLA-4, is a common receptor of nodal immune checkpoint, participating in tumor immune response and tumor immune escape (5–7). So far, most studies on LAG-3 have mainly emphasized its role in T-cell dysfunction and its negative regulatory role in tumor immune response. However, the role of LAG-3 in the tumor microenvironment is not limited to T cells. LAG-3 interacts with a variety of other immune cells, including dendritic cells (DCs) and natural killer (NK) cells, to regulate tumor immune response (8, 9). Although the physiological function of LAG-3 is not well understood, the immune target inhibitors of LAG-3 have shown encouraging properties. In a randomized trial, the phase II/III study revealed that the anti-LAG-3 therapeutic relatlimab, when used alongside nivolumab (anti-PD-1), led to a 12-month progression-free survival (PFS) rate of 47.7% in melanoma patients. This was in contrast to the 36% PFS achieved with nivolumab alone (10). The approval for the combinational therapy of relatlimab and nivolumab was granted by the Food and Drug Administration (FDA) in 2022 for treating unresectable or metastatic melanoma (11).

Considering the crucial clinical relevance and effectiveness of focusing on LAG-3, it is essential to gain additional knowledge on the structural biology, interactions, and signaling pathways associated with LAG-3. This article provides an overview of the LAG-3 structure, its ligands, cell-specific functions, and the outcomes of clinical studies involving LAG-3-targeting agents. The aim is to offer valuable perspectives for investigating the underlying mechanisms of LAG-3 in cancer treatment.

## 2 Structure of LAG-3

**Figure 1:**
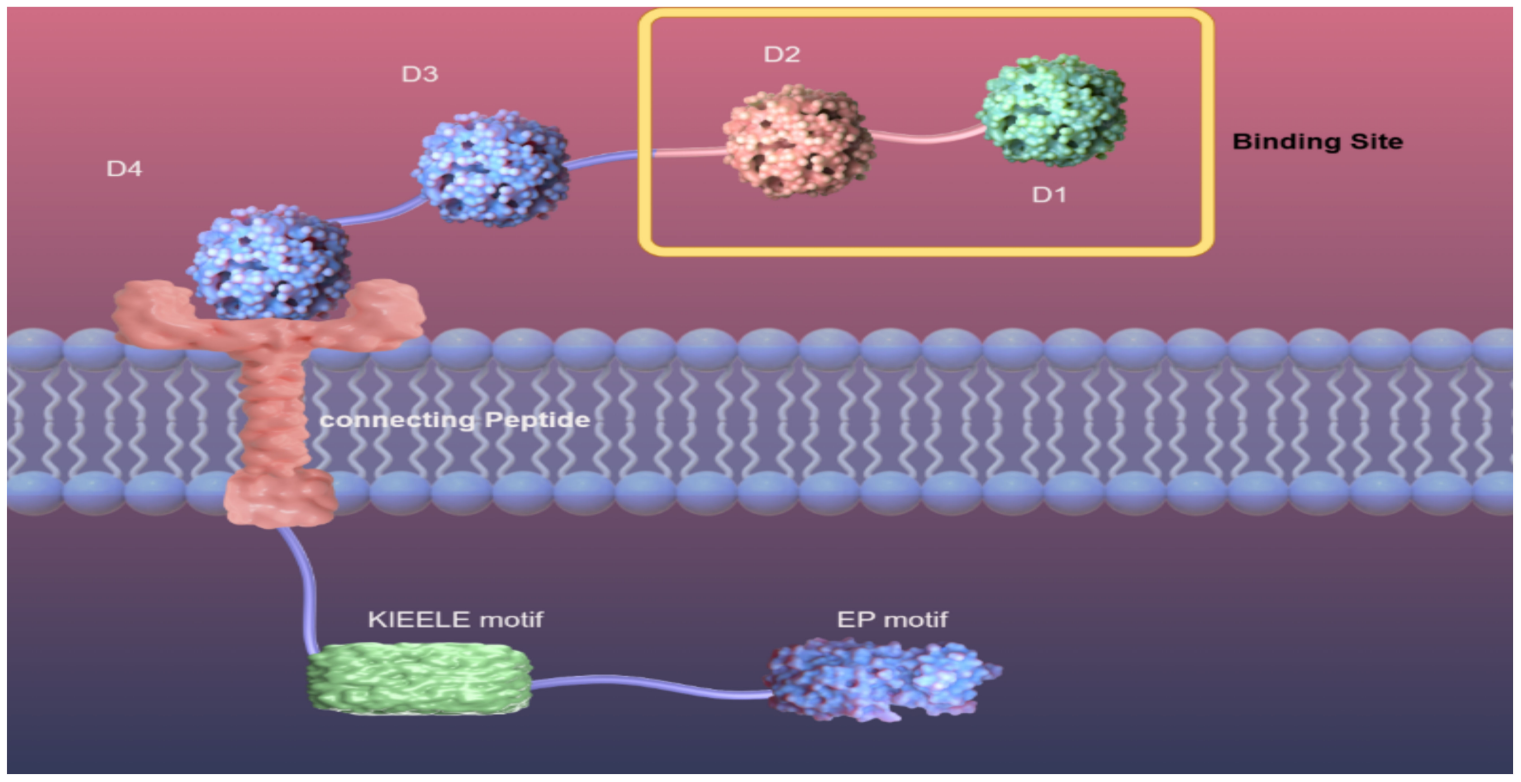
Structure of LAG-3. Diagram of LAG-3 on the surface of a cell membrane. Extracellular region of LAG-3: D1, D2, D3, D4, among which D1 and D2 are binding sites. Transmembrane region and intracellular region of LAG-3: connection peptide, a highly conserved KIEELE motif, EP motif.

LAG-3 (gene 3 lymphocyte-activation), also calledCD223, is a type I transmembrane protein consisting of more than 500 amino acids and weighing 70 kDa. The structure of LAG-3 consists of three parts, including the extracellular region, the transmembrane region, and the intracellular region. The extracellular region consists of four immunoglobulin-like domains, of which the D1 domain contains a proline-rich ring structure and an unusual intrachain disulfide bridge. The D1 domain is species-specific and is known as the V immunoglobulin superfamily, while the D2, D3, and D4 regions belong to the C2 IgSF (**Figure 1**. structure of LAG-3) (12, 13). The transmembrane-intracellular region consists of a potential serine phosphorylation site (S454), a highly conserved KIEELE motif, and a glutamate-proline repeat sequence. The serine phosphorylation site is the action site of tyrosine kinase. The repeat sequence of glutamate-proline, termed the EP motif, plays a key role in intracellular signal transduction (4). Deleting the EP motif or introducing the S454 mutation showed minimal impact on LAG-3 function in both CD3+ and CD4+ T cells. In contrast, eliminating the KIEELE motif in the mutant resulted in a complete loss of normal function. This indicates that the conserved KIEELE motif is crucial for maintaining the proper function of LAG-3 (14).

Fascinatingly, in their research, OKAZAKI et al. (15) discovered that eliminating the KIEELE motif did not abolish the suppressive role of LAG-3. LAG-3 mediates intracellular negative inhibition signaling through two distinct mechanisms that rely on the FXXL motif in the proximal region of the membrane and the EP repeat sequence at the C-terminus.

## 3 LAG-3 ligands

### 3.1 MCH II

**Figure 2:**
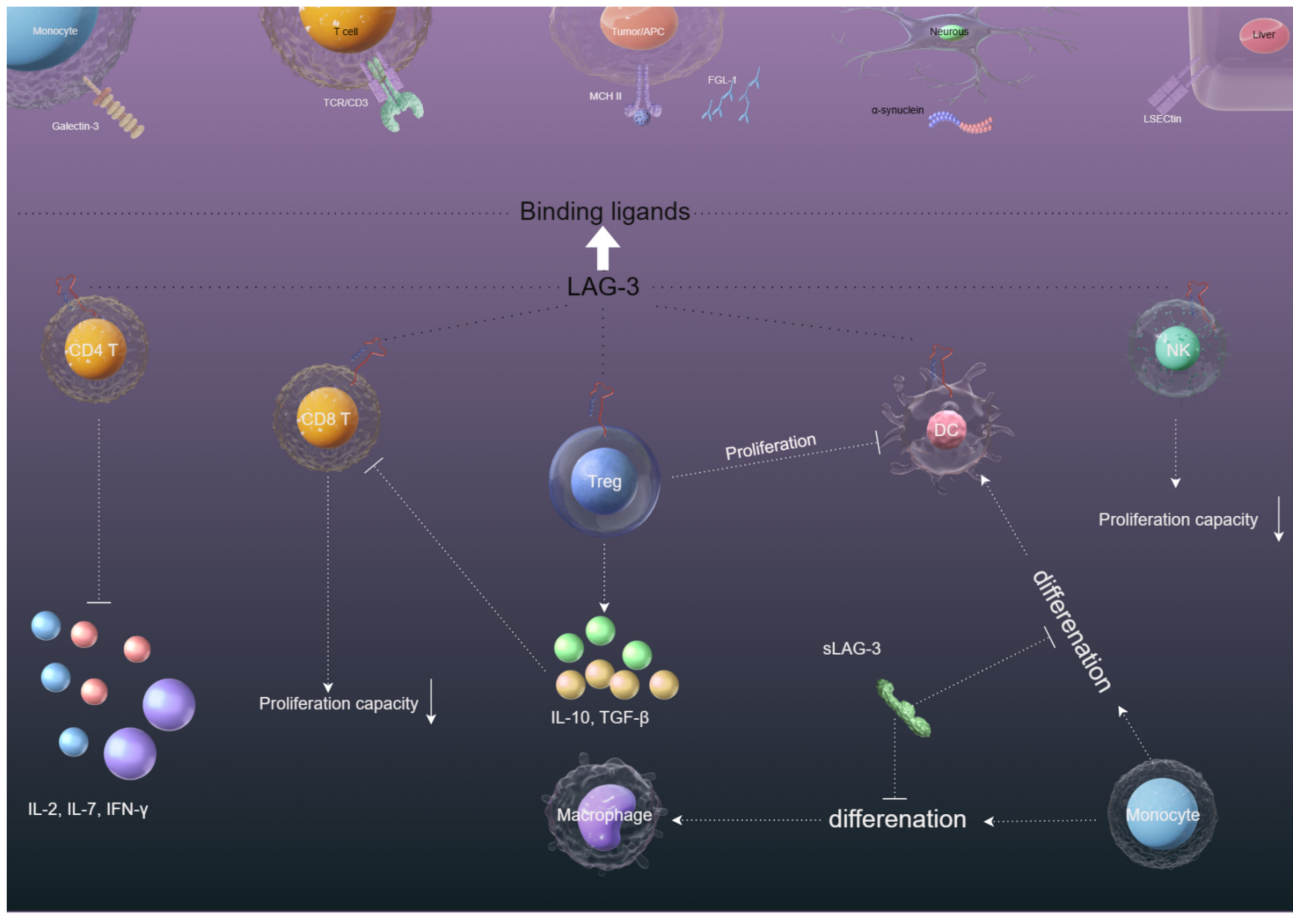
The immunosuppression mechanisms of LAG-3 in the tumor microenvironment. (1) The interaction between LAG-3 and MHC-II on CD4+ cells and tumor cells hinders CD4+ T cell proliferation and cytokine secretion, potentially aiding in tumor cell survival. (2) LAG-3 interaction with Galectin-3/LSECtin/FGL-1 on CD8+/NK cells in the tumor microenvironment suppresses CD8+/NK cell proliferation and cytotoxicity. (3) The binding of LAG-3 with MHC-II on Tregs and tumor cells/DCs enhances the stability and immunosuppressive function of Tregs while compromising DC maturation and immunostimulatory abilities through downstream MHC-II signaling. (4) The presence of sLAG-3 in the tumor microenvironment can disrupt the antigen presentation function of monocyte-derived DCs and impede the differentiation of monocytes into DCs.

Although LAG-3 and CD4 are structurally similar inhibitory surface molecules, they share less than 20% homology at the amino acid level. Similar to CD4, LAG-3 binds to major histocompatibility complex II (MHC II) to negatively regulate T cells, maintain immune system homeostasis, and promote tumor immune escape (**Figure 2**), but with a much stronger affinity to CD4 (4, 16). The binding part of LAG-3 is divided into four domains, of which D1 and D2, alone, are capable of binding MHC II (17). Takumi Maruhashi et al. identified that LAG-3 did not universally recognize MHC II, but selectively recognized stable peptide MHC II (pMHC II) complexes. In addition, LAG-3 did not directly interfere with interactions between the CD4 and MHC II. Instead, LAG-3 preferentially suppressed T cells responsive to stable pMHC II by transducing inhibitory signals via its intracellular region (18). The selective binding of LAG-3 to pMHC II may be related to the molecular mechanism of LAG-3-mediated inhibition.

### 3.2 LSECtin and Gal-3

Interestingly, LAG-3 regulates the proliferation of CD8 T cells without involvement in MHC II, which has led to the search for other LAG-3 ligands (19). Liver and lymph node sinusoidal endothelial cell C-type lectin (LSECtin), which belongs to the C-type lectin receptor superfamily, is a type II transmembrane protein that is highly expressed in the liver and lymph node (20). Feng et al. reported that LSECtin inhibits the proliferation of effector T cells by down-regulating the cell cycle kinases (CDK2, CDK4, and CDK6). LSECtin, expressed in melanoma, interacts with LAG-3 (**Figure 2**) to inhibit IFN-γ secretion by effector T cells, thus promoting tumor growth (21). Although these reports provide us with evidence that LSECtin may be a potential ligand for LAG-3, the mediated regulatory role between LAG-3 and LSECtin is not well understood.

Galactosidin-3 (Gal-3) is a galactoside-binding soluble lectin that is widely distributed in different types of cells and tissues and involved in a variety of biological processes under physiological and pathological conditions, including tumor transformation and metastasis, and immune response (22). Gal-3 has been reported to mediate anti-tumor immune responses by inhibiting CD8+ T cells with LAG-3 and inhibiting the expansion of plasmacytoid dendritic cells (23) (**Figure 2**). Targeting LAG-3/Gal-3 therapy overcomes immunosuppression and enhances anti-tumor response in endometrial cancer (24), multiple myeloma (25), and vulvar squamous neoplasia (26). These reports provide evidence for Gal-3 as a potential ligand of LAG-3.

However, the studies on lectin ligands in LAG-3 are insufficient, and further verification of lectin expression under physiological and pathological conditions and exploration of downstream signaling pathways of LAG-3/Gal-3 and LAG-3/LSECtin interaction are still needed.

### 3.3 FGL1

Fibrinogen-like protein 1 (FGL1) is a fibrinogen secreted by hepatocytes, with differential tumor-specific and site-specific expression (27). The LAG-3 and FGL1 interaction sites are the D1 of LAG3 and the C-terminal fibrinogen-like domain of FGL1 (28). Wang et al. (29) demonstrated that FGL1 is a major immunosuppressive ligand of LAG-3 by using genome-scale receptor arrays and flow cytometry. FGL1 inhibits antigen-specific T-cell activation and deletion of FGL1 in mice promotes T-cell immunity (**Figure 2**). High expression of FGL1 in human plasma is associated with poor prognosis and resistance to anti-PD-1/B7-H1 therapy. To explore the downstream signaling pathway of LAG-3/FGL1 interaction, Jianchu Wang et al. found that oxysophocarpine inhibits FGL1 expression by blocking the IL-6-associated JAK2/STAT3 signaling pathway, sensitizing CD8 T cells to LAG-3 immunotherapy of HCC *in vivo* and *in vitro (* 30). The interaction between the FGL-1 in the cytoplasm of tumor cells interacts with LAG-3 on the surface of various lymphocyte cells and whether other molecular signals are involved in this process remain to be determined. In addition, FGL-1 binds to human LAG-3 and mouse LAG-3 through different molecular surfaces, but how the three interact with each other remains to be explored. In addition, FGL-1 binds to human LAG-3 and mouse LAG-3 via different molecular surfaces (28), but how the three interact with each other remains to be explored. Therefore, FGL-1 is a very potential LAG-3 ligand, and in-depth exploration of the internal pathway of FGL-1/LAG-3 is conducive to further elucidating the inhibitory effect of LAG3/FGL1 on tumors.

### 3.4 α-synuclein

α-synuclein is mainly expressed in neurons, the heart, muscles, and other tissues.

LAG-3 can mediate the spread of α-synuclein fibrils between neurons and affect its endocytosis and intercellular transmission, contributing to Parkinson’s disease (31) (**Figure 2**). Contradictory conclusions have been reported that LAG-3 is not expressed in human and murine neurons and does not modulate α-synucleinopathies (32). However, we cannot deny that α-synuclein/LAG-3 interacts under pathological conditions, for example, LAG-3 can be significantly expressed in brain gliomas (33). Because of whether α-synuclein can be a potential ligand for LAG-3, further study is needed.

### 3.5 T cell receptor/CD3

LAG-3 can also bind to the TCR/CD3 complex in CD4+ and CD8+ T cells in the absence of MHC II (classical ligand), which suggests the TCR/CD3 complex is a substitute ligand for LAG-3 (34). The study also demonstrated that the EP motif of LAG-3 reduced pH at immune synapses and caused tyrosine kinase Lck to dissociate from CD4 or CD8 co-receptors, inhibiting TCR signaling and T cell activation (34) (**Figure 2**). However, the necessary conditions for the interaction of LAG-3 with TCR/CD3 have not been reported, nor is it clear where LAG-3 interacts with TCR/CD3.

Blocking the traditional combination of LAG-3 and MHCII is currently the primary focus of most drug research. Nevertheless, the interaction between additional receptors like FGL-1 and LSECtin with LAG-3 represents a distinct regulatory pathway that operates independently of MHCII and LAG-3. In the future, the development of targeted drugs aimed at blocking these pathways could enhance the effectiveness of targeted therapies.

## 4 The specific function of LAG-3 expression on different cells

### 4.1 LAG-3 and T cells

Like PD-1 and CTLA-4, continuous tumor-associated antigens exposure can result in high and sustained expression of LAG-3 on CD4+ and CD8+ T cells, which negatively regulate T cell expansion and lead to immune disorders, mainly manifested as T-cell exhaustion (35) (**Figure 2**). Workman et al. (36) found that LAG-3-deficient mice amplified more T cells. Adoptive transfer of purified CD4+ and CD8+ T cells to T-cell-deficient mice showed significant expansion of CD4+ and CD8+ T cells in the spleens of LAG-3-deficient mice. To further study whether LAG-3 directly inhibits CD8+ T cells, GROSSO et al. found CD8+ T-cell accumulation in the prostate gland of LAG-3 blocked mice after using the CD4-depleting GK1.5 antibody to consume 95% of CD4+ T cells. In this system, LAG-3 plays a direct role in CD8+ T cells independent of its role in CD4+ cells (6). Blocking LAG-3 can significantly restore CD4+/CD8+ T cell functions (37–39). Although immunotherapies of LAG-3-targeting are currently in clinical trials, how LAG3 inhibits T cell function remains unclear. In general, T cell activation depends on homologous recognition of MHC on the antigen-presenting cells (APCs) surface by TCR, and then transfers the antigen signal to the intracellular immune-receptor tyrosine-based activation motifs (ITAM) region via CD3, thus opening the immune signaling pathway of T cells. Clifford Guy et al. found that LAG-3 moved to immune synapses and associated with TCR-CD3 complex in CD4+ and CD8+ T cells, without binding to MHC II. Mechanistically, the EP motif in the LAG-3 cytoplasmic tail disrupts the interaction of tyrosine kinase Lck and CD4 or CD8 co-receptors, resulting in loss of co-receptor-TCR signaling and limited T cell activation (34). The reasons for these results are mainly related to the unique characteristics of EP motif: (1) EP motif containing a large number of glutamic acid residues reduces the local pH of immune synapses formed by TCR/CD3 and CD4/CD8, disrupting the interaction of tyrosine kinase Lck and CD4/CD8 co-receptors; (2) The EP motif binds the Zn^2+^ that is required for tyrosine kinase Lck and CD4/CD8 co-receptors interactions. Collectively, these features of the EP motif disrupt co-receptor–Lck function, limiting CD3ϵ and ZAP70 phosphorylation and downstream TCR signaling.

Overexpression of LAG-3 in regulatory T cell (Treg) populations has been proven to contribute to their immunosuppressive activity. Huang et al. (7) found that the negative regulatory functions of Tregs were significantly downregulated in LAG-3 deficient mice. Blocking LAG-3 can cause the loss of the inhibitory function of Tregs. However, we have a limited understanding of the endogenous signaling pathway of how LAG-3 mediates the immunosuppressive function of Tregs. Some findings have been made, such as LAG-3 can modulate signal transduction in Tregs and sensitivity to Treg inhibition by downregulating signal transducer and activator of transcription 5 (STAT5). In addition, LAG-3 signaling can increase the differentiation of Foxp3+Treg. Blocking the LAG-3 can reduce the induction of Foxp3+Treg and lead to reduced inhibition and increased CD4+T cell expansion (40, 41). IL-27 has been reported to promote the expression of LAG-3 on Tregs and thus enhance the immunosuppressive function of Tregs in a model for inflammatory bowel disease in humans (42). CD4^+^CD25^-^LAG3^+^ regulatory T cells (LAG3^+^ Treg) are regulated by early growth response gene 2 (Egr2), a zinc-finger transcription factor required for the induction of T-cell anergy. LAG3^+^ Tregs produce large amounts of TGF-β3 in an Egr2- and Fas-dependent manner to inhibit humoral responses (43).

Although the study of LAG-3 interaction with Tregs has brought us some discoveries, a deeper understanding of how LAG-3 systematically affects the functions of T cells is required.

### 4.2 LAG-3 and DCs

DCs, including myeloid DCs and plasmacytoid dendritic cells (pDCs), have the function of antigen presentation and activating lymphocytes to participate in specific immune responses. Workman et al. (44) demonstrated for the first time that LAG-3 can also be expressed on pDCs. By real-time PCR detection, LAG-3 expression in pDCs was ten times that of activated T cells. Activated pDCs produce sLAG-3 five times as many as activated T cells. LAG-3-deficient pDCs proliferate and expand more than wild-type pDCs *in vivo*.

LAG-3 expressed on activated T cells can activate and mature DCs by specific binding to MHC II expressed on immature DCs, and migrate to secondary lymphatic vessels to initiate T-cell activation, which simultaneously produces cytokines such as IL-12 and TNF-α to promote T-cell proliferation and T helper cell 1 (Th1) responses (45–47) (**Figure 2**). However, we have a limited understanding of the downstream signaling pathways of the binding of LAG-3 and MHC II to induce monocytes to mature DCs. Susanne Andreae and colleagues demonstrate that the interaction between MHCII and LAG-3 leads to prompt phosphorylation of PLCγ2 and p72syk proteins, along with activation of PI3K/Akt, ERK1/2, and p38 MAPK signaling pathways. These events are believed to contribute to the stimulation of DC maturation by LAG-3 (8). On the contrary, Buisson et al. (48) demonstrated that sLAG-3 reduced the differentiation of monocytes to macrophages in the presence of granulocyte-macrophage colony-stimulating factors (GM-CSF) and the differentiation of monocytes to dendritic cells in the presence of GM-CSF and IL-4, thus limiting the intensity of the ongoing T cell immune response. The mechanisms that LAG-3 regulates the production of macrophages or DCs *in vivo* are poorly understood and need further study.

### 4.3 LAG-3 and NK cells

While NK cells do express LAG-3 (**Figure 2**), the exact function of LAG-3 in NK cell regulation remains unclear. Miyazaki et al. (49) found that the killing effect of NK cells on tumor lesions was weakened or even disappeared when knockout the LAG-3 gene in mice. However, the NK cells of humans showed the opposite result. Huard et al. (50) showed blocking LAG-3 did not affect the natural killing function of NK cells on target cells. Neither antibodies that block the LAG-3 pathway nor soluble recombinant protein LAG-3-Ig that binds to MHC II have any effect on the killing ability of NK cells. Wiskott-Aldrich syndrome protein deficiency is associated with increased cancer susceptibility, possibly due to reduced antitumor capacity of NK cells and DCs. Wiskott-Aldrich syndrome protein knockout NK cells exhibit cellular exhaustion and NK cell memory associated with increased LAG-3 expression (51–53). Judging from a large number of experimental results, there seems to be a certain connection between LAG-3 and NK cells. Further research is needed on the reasons why opposite results are obtained in the interaction between LAG-3 and NK cells in animal experiments and human experiments.

## 5 Advances in drugs targeting LAG⁃3

Up to now, three forms of LAG-3-targeting drugs have been developed: monoclonal antibodies, bispecific antibodies, and fusion proteins. The results of multiple relevant clinical trials have demonstrated the considerable efficacy and safety of LAG-3-targeting drugs. It also has a good synergistic effect with inhibitors targeting PD-1 and CTLA-4, which can significantly improve the clinical response rate of patients. The following summarizes the clinical efficacy, indications, and further research directions of some of the currently rapidly developing targeted drugs.

### 5.1 Monospecific antibodies of LAG-3

#### 5.1.1 Relatlimab

Relitlimab, an immunoglobulin G4 (IgG4) developed as a potent LAG-3 antagonist, selectively blocks the interaction of LAG-3 with its ligands MHCII and fibrinogen-like protein-1, enhancing TCR signaling and cytokine secretion in activated T cells (54). Ascierto et al. (55) conducted a phase I/II clinical trial (NCT01968109) on 68 melanoma patients unresponsive to previous anti-PD-1/PD-L1 treatments, showing that those with LAG-3-expressing tumors had a higher response rate when treated with the combination of relatlimab and nivolumab, with a safety profile similar to nivolumab alone (**Table 1**). In neoadjuvant therapy for resectable head and neck squamous cell carcinoma (HNSCC), the combination of relatlimab and nivolumab demonstrated safety and promising pathological responses compared to nivolumab monotherapy, highlighting emerging antitumor CD8+ T cell populations and targetable pathways in responder patients (**Table 1**) (56). A phase II/III trial (NCT03470922) evaluating the combination versus nivolumab alone in advanced melanoma showed a median progression-free survival (mPFS) of 10.1 months with the combination versus 4.6 months with nivolumab alone, indicating a greater benefit in progression-free survival with dual inhibition of LAG-3 and PD-1 in patients with metastatic or unresectable melanoma (**Table 1**) (10). The FDA approved a fixed-dose combination of relatlimab and nivolumab for adults and children with unresectable or metastatic melanoma on March 18, 2022 (75).

**Table 1:** LAG-3 immunotherapy clinical trial (https://www.ClinicalTrials.gov).

Drugs	Drug form	Target	Trial identifier	Cohort	Patient group	Status	Phase	Results	Reference
**Relatlimab- Nivolumab**	IgG4 McAb	LAG-3; PD-1	NCT01968109	Relatlimab- Nivolumab (n=68)	Melanoma	Active, not recruiting	I/II	In 61 efficacy-evaluable patients, ORR was 11.5% (1 CR, 6 PR); DCR was 49%. Median DOR was not reached.	(55)
**Relatlimab- Nivolumab**	IgG4 McAb	LAG-3; PD-1	NCT04080804	Relatlimab- Nivolumab (n=13) v. s. Nivolumab- Ipilimumab (n=10) v.s. Nivolumab (n=10)	HNSCC	Recruiting	II	41 patients have been enrolled, with 33 evaluable for this analysis. In the relatlimab- nivolumab (n=13)/nivolumab- Ipilimumab (n=10)/nivolumab (n=10) groups, 1/0/0 patients achieved PR, 10/5/8 patients remained SD, 2/5/2 patients developed PD (RECIST). 7/3/4 patients had a minor partial pathological response (10- 49%), 2/2/0 patients had a partial pathological response (50- 90%), 1/1/0 patients had a major pathological response (> 90%), and 1/0/0 patients had complete pathological.	(56)
**Relatlimab- Nivolumab**	IgG4 McAb	LAG-3; PD-1	NCT03470922	Relatlimab- Nivolumab (n=355) v. s. Nivolumab (n=359)	Melanoma	Active, not recruiting	II/III	Median PFS (relatlimab-nivolumab v. s. nivolumab): 10.1 months v.s. 4.6 months; PFS at 12 months (relatlimab–nivolumab v.s. nivolumab): 47.7% v.s. 36.0%; The ratio of grade 3 or 4 TRAEs (relatlimab-nivolumab v. s. nivolumab): 18.9% v.s. 9.7%.	(10)
**Sym022**	IgG4 McAb	LAG-3	NCT03489369; NCT03311412; NCT03489343	Sym021- Sym022 (n=20) v.s. Sym021 (n=17) v.s. Sym022 (n=15)	Metastatic cancer; Solid Tumor; Lymphoma	Completed	I	In the Sym021- Sym022/Sym021/Sym022 arms, 0/1/0 achieved CR and 1/1/1 achieved PR.	(57)
**Ieramilimab(LAG525)- Spartalizumab (PDR001)**	IgG4 McAb	LAG-3; PD-1	NCT03365791	LAG525- PDR001 (n=72)	Ovarian adenocarcinoma; GC; DLBCL; SCLC; NET; Prostate; Sarcoma	Completed	II	NET, SCLC, and DLBCL cohorts all met the expansion criteria with the posterior probability that clinical benefit exceeds historical control of 0.971, 0.975, and 0.804 respectively. Clinical benefit rate at 24 weeks were as follows; NET: 0.86 (6/7), SCLC: 0.27 (4/15), DLBCL: 0.43 (3/7).	(58)
**Ieramilimab (LAG525)**	IgG4 McAb	LAG-3	NCT02460224	LAG525- PDR001 (n=99) v.s. LAG525 (n=115)	Advanced solid tumors	Active, not recruiting	I/II	LAG525- spartalizumab led to durable RECIST responses (11 PR, 1 CR) in a variety of solid tumors, including mesothelioma (2/8 patients) and triple-negative breast cancer (2/5 patients).	(59)
**Ieramilimab(LAG525)**	IgG4 McAb	LAG-3	NCT03499899	LAG525- PDR001 (n=20) v.s. LAG525- PDR001- carbo (n=34) v.s. LAG525- carbo (n=34)	TNBC	Active, not recruiting	II	ORR (LAG525- PDR001 v.s. LAG525- PDR001- Carboplatin v.s. LAG525- Carboplatin):7.1% v.s. 32.5% v.s. 18.4%; DOR (LAG525- PDR001 v.s. LAG525- PDR001- Carboplatin v.s. LAG525- Carboplatin):4.9 months v.s. 13.6 months v.s. 12.6 months.	(60)
**INCAGN02385**	IgG1-Fc	LAG-3	NCT03538028	INCAGN02385 (n=22)	GC; ovarian cancer; HCC; NSCLC; melanoma; Urothelial carcinoma	Completed	I	The doses of INCAGN02385 ≥250 mg led to trough LAG-3 receptor occupancy of ≥90% in peripheral blood and increased markers for CD4+ T-cell proliferation. DCR was 27%.	(61)
**LBL-007 and Toripalimab**	IgG4 McAb	LAG-3; PD-1	NCT04640545	Part A: LBL-007- Toripalimab (n=68); Part B: LBL-007- Toripalimab- Axitinib (n=11)	Melanoma	Recruiting	I	Part A: ORR was 45.4% (including 4 mucosal and 1 acral), DCR was 72.7%, and median PFS was 5.5 months. Part B: ORR was 45.4%, DCR was 72.7%, and mPFS was 5.5 months	(62)
**Fianlimab (REGN3767)-Cemiplimab**	IgG4 McAb	LAG-3	NCT03005782	REGN3767- Cemiplimab (n=42) v.s. REGN3767 (n=27)	Malignancies	Active, not recruiting	I	The best response was stable disease in 11 patients (RECIST 1.1) in the REGN3767 monotherapy group (n=27); 2 (both small cell lung cancer) combination group patients and 2 (endometrial cancer and cutaneous squamous cell carcinoma) of 12 additional patients who crossed over from monotherapy group to combination group had partial responses; pharmacokinetics: R3767 concentrations in serum increased in a dose-dependent manner and were unaffected by combination.	(63)
**Fianlimab (REGN3767)-Cemiplimab**	IgG4 McAb	LAG-3	NCT03005782	REGN3767-Cemiplimab: anti–PD-(L)1 naive group (n=33) v.s. anti–PD-(L)1 experienced group (n=15)	Advanced melanoma	Active, not recruiting	I	By investigator assessment, ORR was 63.6% (3 CRs and 18 PRs) for patients who had no prior anti–PD-(L)1 treatment and 13.3% (1 CR and 1 PR) for anti–PD-(L)1 experienced patients; mPFS and mDOR for the patients who had no prior anti–PD-(L)1 treatment cohort have not been reached.	(64)
**Miptenalimab (BI 754111)- ezabenlimab (BI 754091)**	IgG4 McAb	LAG-3; PD-1	NCT03433898	Cohort A: patients with gastric/gastroesophageal junction cancer (n=36) v.s. Cohort B: esophageal cancer (n=37)	Neoplasms	Completed	I	Confirmed PR was observed in 4/7 patients in cohorts A/B; ORR was 11% and 19%. SD was observed in 10/8 (28/22%) patients in cohorts A/B and DCR was 39/41%.	(65)
**Tebotelimab (MGD013)**	BsAb	LAG-3; PD-1	NCT03219268	MGD013: 50 patients were treated in dose-escalation, and 157 patients in dose-expansion.	metastatic neoplasms	Completed	I	Among 41 response-evaluable dose-escalation patients, 3 patients were observed confirmed PR (triple negative breast cancer, mesothelioma, GC; RECIST 1.1), while 21 patients had SD. Among select expansion cohorts, PRs have been observed in epithelial ovarian cancer (n=2/15) and TNBC (n=2). SD has been observed in epithelial ovarian cancer (n=7/15) and TNBC (n=5/14).	(66)
**Tebotelimab (MGD013)-Niraparib**	BsAb	LAG-3; PD-1	NCT04178460	MGD013- Niraparib (n=27)	GC	Terminated	I	In patients with target lesions on the recommended phase II dose (tebotelimab 600 mg / 2 weeks plus niraparib / individualized starting doses once daily; n=19), one confirmed PR (RECIST v1.1) was observed and 9 patients had SD, with a 5.3% ORR and a 52.6% dcr. Inpatients on recommended phase II dose (n=21), median PFS and median OS were 2.7 and 6.5 months, respectively, after a median follow-up of 7.7 months.	(67)
**RO7247669**	BsAb	LAG-3; PD-1	NCT04140500	RO7247669 (n=35)	NSCLC; metastatic melanoma	Recruiting	I/II	ORR was 17.1 %, and DCR was 51.4 %. Responses have been observed in checkpoint inhibitors naive patients (4/23) as well as in checkpoint inhibitors experienced patients (2/12).	(68)
**Eftilagimod alpha (IMP321)- Avelumab**	Soluble protein	LAG-3; PD-L1	NCT03252938	Cohort 1: Avelumab-IMP321 6mg (n=6) v.s. Cohort 2: Avelumab+IMP321 30mg (n=6)	Solid Tumors; Peritoneal carcinomatosis	Recruiting	I	As of September 2020, 12 patients with advanced solid tumors were treated with IMP321 and avelumab, 4 patients have achieved PR and 3 patients have progressed. 2 patients progressed clinically and 3 patients did not undergo tumor evaluation.	(69)
**Eftilagimod alpha (IMP321)- Paclitaxel**	Soluble protein	LAG-3	NCT02614833	Cohort 1: Paclitaxel-IMP321 6mg (n=6) v.s. Cohort 2: Paclitaxel- IMP321 30mg (n=9)	Adenocarcinoma breast (Stage IV)	Completed	II	An increased number of circulating monocytes, dendritic cells, and increased activation were observed with the treatment of IMP321. Seven patients (47 %) had a PR according to RECIST 1.1 (mean duration of 9 months). The DCR was 87 %.	(70)
**Eftilagimod alpha (IMP321) Pembrolizumab**	Soluble protein	LAG-3; PD-1	NCT02676869	IMP321-Pembrolizumab (n=18)	Melanoma (Stage III-IV)	Completed	I	16 patients were eligible for response evaluation. In 8 (50 %) patients, a tumor reduction was observed. This includes one patient with a confirmed CR after initial progression on pembrolizumab monotherapy.	(71)
**Eftilagimod alpha (IMP321)- Pembrolizumab**	Soluble protein	LAG-3; PD-1	NCT03625323	IMP321+Pembrolizumab (n=38)	HNSCC	Active, not recruiting	II	35 patients were evaluated for response (cut-off Jan 2021) with 4 (11 %) patients showing CR, 7 (20 %) patients PR, 3 (9 %) patients SD, 16 (46 %) patients PD with 5 (14 %) patients being not evaluable (iRECIST). ORR was 31.4 % and DCR was 40 %. Median PFS was 2.1 months and 35 % were progression-free at 6 months. The median OS was 12.6 months.	(72)
**Eftilagimod alpha (IMP321)- Pembrolizumab**	Soluble protein	LAG-3; PD-1	–	IMP321+Pembrolizumab (n=24): IMP321 at doses 1 mg, 6 mg, or 30 mg/injection for up to 6 months (part A) and 30 mg/injection for up to 12 months (part B)	Melanoma	–	–	Treatment induced an increase in activated CD8 and CD4 T cell counts, and in some of the soluble biomarkers, particularly interferon (IFN)-γ, a Th1 signature cytokine. An ORR of 33% was observed in patients partly with pembrolizumab-refractory of part A and an ORR of 50% was observed in patients with PD-1 naïve of part B.	(73)
**FS-118**	BsAb	LAG-3; PD-L1	NCT03440437	FS-118 (n=43)	Advanced Cancer; Metastatic Cancer; HNSCC	Active, not recruiting	I/II	The DCR was 46.5%; Pharmacodynamic activity was prolonged throughout dosing as demonstrated by sustained elevation of soluble LAG-3 and increased peripheral effector cells.	(74)

BsAb, bispecific antibody; McAb, monoclonal antibody; HNSCC, head and neck squamous cell carcinoma; NSCLC, non-small cell lung cancer; SCLC, small cell lung cancer; HCC, hepatocellular carcinoma; GC, gastric cancer; TNBC, triple-negative breast cancer; NET, neuroendocrine tumor; DLBCL, diffuse large B-cell lymphoma; PR, partial response; CR, complete response; SD, stable disease; PD, progressive disease; DCR, disease control rate; ORR, objective response rate; OS, overall survival; PFS, progression-free survival; DOR, duration of overall response.

#### 5.1.2 LBL-007

LBL-007, a novel anti-LAG-3 antibody derived from a human antibody phage display library, specifically targets the LAG-3 antigen on activated T cells, enhancing interleukin-2 secretion. It exhibits superior internalization through endocytosis compared to the relatlimab analog. LBL-007 effectively hinders the interaction between LAG-3 and MHCII, thereby blocking downstream signaling. In a mouse model with colorectal cancer cells, combining LBL-007 with an anti-PD-1 inhibitor demonstrated significant inhibition of tumor growth (38). In a clinical trial (NCT04640545) (**Table 1**), 55 efficacy evaluable patients with advanced melanoma received LBL-007 in conjunction with toripalimab, resulting in an ORR of 23.6%, DCR of 58.2%, and mPFS of 5.7 months. In another part of the study, 11 patients treated with LBL-007 alongside toripalimab and axitinib achieved an ORR of 45.4%, DCR of 72.7%, and mPFS of 5.5 months. Notably, 27.9% of patients in the former part and 45.5% in the latter part experienced grade ≥ 3 treatment-related adverse events (TRAEs). The combination of LBL-007 and toripalimab exhibits promising antitumor effects with a manageable safety profile in treatment-naive melanoma patients (62).

#### 5.1.3 Ieramilimab

In the phase I/II study involving 255 patients with advanced malignancies, the use of ieramilimab (LAG525) as a single agent or in combination with spartalizumab resulted in varying levels of treatment-related adverse events (TRAEs). The majority of patients experienced TRAEs such as fatigue, gastrointestinal reactions, and skin disorders. Additionally, a small percentage of patients in both groups achieved SD for 6 months or longer, with complete remission seen in 3 patients and PR in 10 patients in the combination group. Overall, leramilimab was well tolerated when used alone or in combination with spartalizumab, showing modest antitumor activity with combination therapy (59). Furthermore, spartalizumab and LAG525 demonstrated promising activity in specific types of tumors such as neuroendocrine tumor (NET), small cell lung cancer (SCLC), and diffuse large B-cell lymphoma (DLBCL) in a phase II study (NCT03365791) (**Table 1**) (58). NCT03499899 evaluated the efficacy of LAG525 in combination with spartalizumab, spartalizumab, and carboplatin, or carboplatin as first- or second-line treatment in patients with advanced triple-negative breast cancer (TNBC). The combination of LAG525 with PDR001 and carboplatin showed the highest objective response rate (ORR) at 32.4% with a response duration of 13.6 months (**Table 1**) (60).

#### 5.1.4 Fianlimab

Fianlimab (REGN3767), a human IgG4 antibody, binds strongly to LAG-3 in both human and monkey species, effectively preventing LAG-3 from interacting with MHCII ligands and reversing its inhibitory effects on T-cell function. Burova et al (76) utilize a humanized PD-1/LAG-3 knock-in mouse model to evaluate the impact of REGN3767 either alone or in combination with REGN2810 on the growth of MC38 tumors *in vivo*. The combination treatment significantly suppressed tumor growth compared to individual treatments with Regn3767 or REGN2810. Analysis of MC38 tumor cells through RNA sequencing and RT-PCR revealed that the combined therapy not only increased antitumor efficacy and induced gene expression alterations not observed with monotherapies but also enhanced immune responses correlated with T cell activation and effector function normally promoted by each antibody alone. Furthermore, treatment of human PD-1xLAG-3 knock-in mice with Regn3767 in combination with cemiplimab (a human anti-PD-1 antibody) demonstrated heightened antitumor effects and facilitated the release of pro-inflammatory factors by tumor-specific T cells, potentially attributed to the disruption of inhibitory signaling mediated by hLAG-3/MHCII in the presence of PD-1/PD-L1 (76, 77). Initial human studies assessing the safety of Regn3767 alone or in conjunction with cemiplimab indicated manageable side effects. Although the challenge of curing many patients remains, promising initial therapeutic responses have been observed (63). In the study (NCT03005782) (**Table 1**), forty-eight participants (thirty-three PD-(L)1 treatment-naive and fifteen anti–PD-(L)1 experienced) with late-stage melanoma received treatment with fianlimab and cemiplimab. According to the evaluator’s review, the overall response rate was 63.6% (three complete responses and eighteen partial responses) for individuals without previous anti–PD-(L)1 therapy and 13.3% (one complete response and one partial response) for those who had received anti–PD-(L)1 treatment. The combined use of fianlimab and cemiplimab exhibited a favorable safety profile and clinical effectiveness, akin to the treatment combining anti-PD-1 and CTLA-4, albeit with lower documented rates of treatment-related side effects (64). Currently, a phase III study (NCT05608291) is underway to compare fianlimab combined with cemiplimab against pembrolizumab in individuals diagnosed with fully removed high-risk melanoma. This trial aims to offer further verification of the effectiveness of utilizing the combination of LAG-3 and PD-1 in the treatment of melanoma (78).

#### 5.1.5 INCAGN02385

INCAGN02385 is a humanized monoclonal antibody of the IgG1κ subtype that has been engineered with an Fc region to enhance its affinity and specificity. This antibody is designed to effectively block the interaction between LAG-3 and its ligands, specifically MHCII, thereby reversing the inhibitory effects of LAG-3 on T-cell function. A recent phase I clinical trial (NCT03538028) (**Table 1**) involving 22 patients with advanced solid tumors demonstrated the favorable safety profile of INCAGN02385. Administration of INCAGN02385 at a dose of ≥250 mg every two weeks resulted in achieving ≥90% LAG-3 receptor occupancy in the peripheral blood, leading to increased levels of markers indicative of CD4+ T cell proliferation (61). Additionally, several monotherapy and combination therapy studies involving INCAGN02385 are currently in progress.

#### 5.1.6 Sym022

Sym022 is a monoclonal antibody that is Fc-inert and specifically targets LAG-3 in humans. It binds strongly to LAG-3 and disrupts the interaction between LAG-3 and MHCII. By modulating T-cell cytokine production, Sym022 effectively inhibits tumor growth *in vivo*. The mechanism of action involves preventing ligand binding and reducing overall levels of LAG-3 on the cell surface through internalization or shedding (79). An ongoing phase I clinical trial with registration number NCT03489369 (**Table 1**) is investigating the safety, tolerability, and potential anti-cancer activity of Sym022 in patients with advanced solid tumors or lymphomas. Among the participants, 15 were given Sym022 alone, while 20 received a combination of Sym022 and an anti-PD-1 antibody. Notably, no immune-related adverse events were observed in the group that received Sym022 alone, and only 4 out of 20 patients experienced such events in the combination therapy group. The results suggest that Sym022, whether used as a monotherapy or in conjunction with PD-1 inhibitors, was well tolerated (**Table 1**) (57). Another clinical trial with registration number NCT04641871 is planned to assess the efficacy of Sym022 in patients with biliary tract cancer and esophageal squamous cell carcinoma who have already undergone first-line chemotherapy.

#### 5.1.7 Encelimab

Encelimab, also known as TSR-033, is an IgG4 monoclonal antibody that exhibits strong binding and selectivity for LAG-3. This antibody was humanized and derived from the collaboration between Tesaro and Anaptysbio, as documented in US patent number 2022135670 (80). The antibody of LAG-3 has been shown to increase T cell activation in various *in vitro* assays, leading to a potential enhancement of immune response. Additionally, in a humanized mouse model of non-small cell lung cancer (NSCLC), combining TSR-033 with TSR-042 resulted in enhanced antitumor efficacy compared to using TSR-042 alone. This combination treatment led to a significant increase in the total number of intratumor T cells, including CD8+ T cells, as well as heightened T cell proliferation. These findings suggest that targeting LAG-3 in combination with anti-PD-1 therapy could be a promising approach for enhancing immune response and improving treatment outcomes in NSCLC (81).

#### 5.1.8 Miptenalimab

BI 754111, also known as Miptenalimab, is one of several anti-LAG-3 antibodies identified in the US2021095020 patent by Boehringer Ingelheim (82). In the MC38 tumor model, the synergistic effect of miptenalimab resulted in a significant enhancement of antitumor efficacy when compared to the use of anti-PD-1 antibody as a standalone treatment. Within an *in vitro* setting simulating antigenic memory T cells expressing PD-1 and LAG-3, there was a notable increase in interferon (IFN)-γ secretion. Specifically, there was a 6.9-fold rise in secretion observed with ezabenlimab (BI 754091; anti-PD-1 antibody) as a monotherapy and a remarkable 13.2-fold increase when ezabenlimab was used in conjunction with BI 754111, in comparison to controls with similar genetic background (83). NCT03156114, NCT03433898, NCT03697304, and NCT03780725 presented safety data on the combination of BI 754111 and BI 754091 in advanced solid tumor patients. The recommended phase II dose of BI 754111 (600 mg) plus BI 754091 (240 mg q3w) was administered to 285 patients. Adverse effects such as fatigue (22.8%), pyrexia (18.6%), and nausea (16.5%) were observed. This indicates that the combination has a well-controlled safety profile (84). In the study, NCT03433898 (**Table 1**), four patients with gastric or gastroesophageal junction cancer/esophageal cancer showed confirmed partial response. The ORR was 11%, with a DCR of 39%. Additionally, 28% and 22% of patients with gastric or gastroesophageal junction cancer/esophageal cancer respectively had SD. The study detected early signals of efficacy in this treatment combination (65).

### 5.2 Soluble LAG-3

Fully developed LAG-3 molecules can split at the cellular membrane, resulting in the creation of the soluble segment P54 (which consists of D1, D2, and D3, known as sLAG-3) and the transmembrane cytoplasmic segment P16 (85). In 2006, Casati et al. (86) discovered that the cooperation between sLAG-3 and MHCII triggers the stimulation of APC to enhance the production and expansion of CD8+ T cells, suggesting that sLAG-3 can rival LAG-3 molecules in binding to MHCII and counteracting the suppressive impact of LAG-3. During clinical trials investigating the function of sLAG-3 in GC, researchers discovered that patients with GC exhibited reduced levels of sLAG-3 in their peripheral blood. Interestingly, elevated sLAG-3 levels were associated with a favorable prognosis for GC. In mouse studies, sLAG-3 was shown to potentially impede tumor cell growth and enhance the production of IL-12 and IFN-γ by CD8+ T cells. Moreover, sLAG-3 administration appeared to enhance the overall survival (OS) and survival rates of GC-afflicted mice (87). In a clinical trial that examined sLAG-3 in patients with NSCLC, sLAG-3 was associated with tumor stage. sLAG-3 levels were significantly higher in stage I-II NSCLC than in stage III-IV NSCLC, which was thought to be related to differences in the cancer immune response in patients with advanced disease. Therefore, improving sLAG-3 levels in patients with advanced NSCLC may be a promising treatment (88).

The 200-kDa dimer of recombinant soluble human LAG-3Ig fusion protein (known as Eftilagimod alpha or IMP321) was generated in Chinese hamster ovary cells by introducing a plasmid that contains the extracellular portion of human LAG-3 connected to the human IgG1 Fc region (89). Eftilagimod alpha activate APCs can lead to CD8+ T cell activation and binding with MHC II molecule subtypes expressed on immature DCs induces the rapid formation of dendritic processes. Furthermore, eftilagimod alpha significantly increases the expression of costimulatory molecules, along with the secretion of IL-12 and tumor necrosis factor (TNF)-α (8, 46, 89, 90). The combination of Eftilagimod alpha and anti-PD-1/PD-L1 inhibitors for solid tumors has shown encouraging therapeutic potential and a controllable safety profile (69, 71). A total of 24 individuals diagnosed with melanoma were treated with pembrolizumab in conjunction with eftilagimod alpha. This treatment resulted in a rise in the number of activated CD8+ and CD4+ T-cells, as well as an increase in certain soluble biomarkers, most notably IFN-γ, which is a cytokine associated with Th1 immunity. The ORR stood at 33% during the dose escalation phase and climbed to 50% during the study’s extension phase. The combination of eftilagimod alpha and pembrolizumab demonstrated promising anti-tumor effects and exhibited a favorable safety profile (**Table 1**) (73). During the clinical trial NCT02614833 (**Table 1**), 15 individuals diagnosed with advanced breast cancer were administered IMP321 alongside paclitaxel. Among the participants, 7 individuals (accounting for 47%) displayed partial response (with an average duration of 9 months) based on RECIST 1.1 criteria. The DCR was determined to be 87%. Furthermore, an elevation in the quantity of circulating monocytes, DCs, and CD8+ T cells, along with an enhanced state of cellular activation, was identified in these subjects. This continual state of cellular response activation was linked to escalated levels of Th1 markers in the bloodstream (70). Encouraging results were also observed with IMP321 and pembrolizumab in the treatment of metastatic head and neck squamous cell carcinoma (HNSCC), 4 (11%) patients showing CR, 7 (20%) patients PR, 3 (9%) patients SD, 16 (46%) patients showing progressive disease (PD) with 5 (14%) patients being not evaluable (iRECIST). ORR was 31.4% and DCR was 40%. Median PFS was 2.1 months and 35% were progression-free at 6 months. Median OS was 12.6 months– Encouraging results were also observed with the combination of IMP321 and pembrolizumab in treating metastatic head and neck squamous cell carcinoma (HNSCC). Among the patients, 4 individuals (11%) achieved CR, 7 patients (20%) showed PR, 3 patients (9%) had SD, and 16 patients (46%) experienced progressive disease (PD). Additionally, 5 patients (14%) were deemed unevaluable based on iRECIST criteria. The ORR was 31.4%, while the DCR was 40%. The median PFS (mPS) was 2.1 months, and 35% of patients remained free from progression at 6 months. The median OS (mOS) was 12.6 months (**Table 1**) (72).

The immunostimulating function of sLAG-3 is crucial in cancer treatment, and the presence of sLAG-3 indicates a positive outlook for certain individuals with tumors.

### 5.3 Bispecific antibodies of LAG-3

While anti-LAG-3 antibodies by themselves showed initial effectiveness against tumors and were deemed safe, the use of LAG-3 therapy alone is frequently linked to limited success rates and faster development of resistance. This is in part due to other immune checkpoint receptors, such as TIM-3, which are commonly present alongside PD-1 in lymphocytes that infiltrate tumors (91, 92). LAG-3 and PD-1 have synergistic effects on the immunosuppression and escape of tumor cells. Huang et al. (93) found that the correlation between LAG-3 and PD-1 enables them to be transported rapidly to immunological synapses, which restricts the signaling of CD8+ T cells and inhibits the antitumor response in mouse ovarian cancer models. Huang et al. (94) found that tumor-free mice with triple blockade of the immune checkpoint pathway of PD-1/CTLA-4/LAG-3 had a significantly higher percentage of survival than those with double blockage of PD-1/CTLA-4. Blocking LAG-3 demonstrated a synergistic effect when combined with PD-1 inhibition. The dual blockade enhanced the regeneration of T cells and the effectiveness against tumors, surpassing the outcomes of LAG-3 therapy alone (95, 96). Therefore, the search for a combination therapy for LAG-3 and other immune checkpoints is promising.

#### 5.3.1 Tebotelimab

Tebotelimab, also known as MGD013, is a tetravalent bispecific protein with a humanized Fc region. It is constructed using monoclonal antibodies targeting LAG-3 and PD-1 (97). MGD013 can specifically bind LAG-3 and PD-1 and block the interaction of PD-1/PD-L1, PD-1/PD-L2, and LAG-3/MHCII, enhancing cytokine secretion and awakening exhausted T-cell function (98). In a first-in-human, open-label, phase I study of MGD013 (NCT03219268) (**Table 1**), the safety, tolerability, and anti-tumor effects of MGD013 were evaluated in patients with advanced solid and hematologic malignancies. Results showed that 59% of patients with assessable efficacy achieved SD or better during dose escalation. Furthermore, some patients with epithelial ovarian cancer and triple-negative breast cancer demonstrated PR in the dose-expansion phase. TRAEs were observed in 70.5% of patients, with fatigue (19%) and nausea (11%) being the most common. The incidence of grade ≥ 3 TRAEs was 23.2% (66). After the MGD013 therapy, the levels of serum IFN-γ saw a notable rise, exceeding 140 times the initial level. Furthermore, an elevation in the populations of circulating CD3+CD8+ and CD3+CD4-CD8- T-cell subsets, along with the associated cytolytic indicators like perforin and granzyme B, were detected in patients with diffuse large B-cell lymphoma (99). In HCC tissues, the expression of LAG-3 has also increased in the vast majority of tumor-infiltrating lymphocytes with positive PD-1 staining, but it was also found that only a single target of LAG-3 was upregulated in a small number of cases, which suggests that some HCC patients may benefit from the inhibition of the LAG-3 pathway rather than the PD-1 pathway (100). LAG-3 immune checkpoints may limit the efficacy of other monotherapies that block HCC targets. In a dose-expansion phase II study (NCT04212221) evaluating the safety and efficacy of tebotelimab (MGD013) in patients with HCC, the ORR of 3.3% for ICI-experienced cohorts (previously treated with ICIs) was significantly lower than the 13.3% for ICI-naive cohorts (not previously treated with ICIs). However, mPFS was 2.4 and 3.1 months for ICI-experienced and ICI-naïve cohorts, respectively, with mOS not reached in both (101). Reasons considered for the unsatisfactory antitumor activities include resistance to multiple previous ICI treatments in these patients, low number of cases, dose selection reasons, drug interactions, etc. Also, in a study of the combination of tebotelimab and niraparib in patients with locally advanced or metastatic GC who failed prior treatments (NCT04178460) (**Table 1**), although this combination demonstrated a manageable safety profile, its antitumor activity was limited, with an ORR of only 5.3% when treated with recommended phase II dose (67). Unlike HCC, higher LAG-3 expression in GC is associated with a better patient prognosis. A study included 385 patients with stage II/III GC, and immunohistochemical analysis revealed that 50.1% of the patients had LAG-3 expression. Survival analysis using Kaplan-Meier demonstrated that patients with gastric cancer who exhibited positive LAG-3 expression at the invasive margin or central region tended to improve overall survival compared to individuals with negative expression (102).

#### 5.3.2 RO7247669

RO7247669 is another bispecific anti-PD-1/LAG-3 antibody similar to MGD013. This antibody can reactivate dysfunctional T cells and overcome LAG3-mediated resistance to ICIs. In a preliminary study involving 35 patients with metastatic solid tumors, the treatment with RO7247669 resulted in an ORR of 17.1% and a DCR of 51.4%. It was found that 17.1% of patients experienced Grade 3 treatment-related adverse events (TRAEs), while there were no Grade 4-5 TRAEs recorded, and no dose-limiting toxicity was observed. Overall, RO7247669 shows promising safety and clinical activity in patients with metastatic solid tumors. The ORR and DCR values indicate a positive response to the treatment, with manageable Grade 3 TRAEs. The absence of Grade 4-5 TRAEs and dose-limiting toxicity further support the safety profile of RO7247669 in this patient population. Further research and larger clinical trials are warranted to fully evaluate the efficacy and safety of this bispecific antibody in a broader patient population (**Table 1**) (68).

#### 5.3.3 IBI323

IBI323 is a human IgG1 bispecific antibody synthesized by IBI110 (anti-LAG-3) and Bi127 (anti-PD-L1) that targets PD-L1 and LAG-1 and has a reduced FC-mediated antibody effect function. IBI323 mediates the bridging of PD-L1+ cells and LAG-3+ cells, exhibiting immunostimulatory activity superior to that of each parent antibody in mixed leukocyte responses. The stronger antitumor activity of IBI323 is associated with an increase in tumor-specific CD8+ and CD4+ T cells compared to each parental antibody in PD-L1/LAG-3 double-knocking mice carrying human PD-L1 knocking to MC38 tumors (103). Shang Hai Pulmonary Hospital is conducting a phase I clinical trial (NCT04916119) to evaluate the safety, efficacy, and pharmacokinetics of IBI in the treatment of advanced malignant tumors.

#### 5.3.4 FS-118

FS-118 is a quadrivalent bispecific antibody targeting LAG-3 and PD-L1 with greater preclinical activity compared to monoclonal antibody combinations. In a murine tumor model, FS-118 decreases LAG-3 expression on tumor-infiltrating lymphocytes (TILs) while raising sLAG-3 levels in mouse serum (104). Simultaneously, higher levels of sLAG-3 were observed in the bloodstream of individuals receiving treatment with FS118. In human T-cell experiments performed in a laboratory setting, FS118-induced elevation of sLAG-3 exceeded that of the individual bispecific constituents combined. In comparison to a stand-alone PD-L1 monoclonal antibody, FS118 amplified the activation of human CD8+ T-cells upon exposure to MHC Class I restricted peptides (105). In the first human trial of FS118 (NCT03440437) (**Table 1**), 43 patients with advanced cancer and PD-L1 resistance received FS-118 monotherapy. During treatment, FS-118 was well tolerated and no serious TRAEs associated with FS-118 were reported. No dose-limiting toxicity was observed and no MTD was achieved. The overall DCR was 46.5% (74). No adverse reactions from FS-118 were detected, thus additional investigations at increased dosages are necessary to evaluate the therapeutic potential in individuals who have developed resistance to anti–PD-L1 treatment.

#### 5.3.5 Bavunalimab

Both CTLA-4 and LAG-3 are co-suppressor receptors of T cells, which are associated with T cell activation and CD8+T lymphocyte failure caused by malignant tumors.

Activation of the CTLA-4 receptor can inhibit the production of IL-2 in CD4+ T-cells, and CTLA-4 blocking indirectly improves the cytotoxicity of NK cells by ensuring an adequate supply of IL-2 to CD4+ T-cells (106). Blocking LAG-3 expression was associated with improved NK cell depletion. In addition, blocking LAG-3 and CTLA-4 on the surface of NK cells has a synergistic effect in increasing the release of IFN-γ and TNF-α (107). Bavunalimab (XmAb841 or XmAb22841) is a bispecific anti-CTLA-4/LAG-3 antibody, which activated T cells in NSG mice to achieve anti-tumor effects (80).

Bispecific antibodies have significant advantages compared to monoclonal antibodies, yet no products in this category have received marketing approval thus far. There is ample opportunity for further research and development in this field. Additionally, exploring a rational combination strategy involving LAG-3 targeted immunotherapy and other targeted drugs, such as chemotherapy and radiotherapy, to optimize clinical efficacy is a promising direction for investigation.

## 6 Conclusion

Currently, most clinical trials of LAG-3 inhibitors have focused on the combination of LAG-3 and PD-1, as this combination has been approved by the FDA and has shown encouraging results in clinical trials of multiple tumor types. However, our understanding of LAG-3 is very limited, and many questions remain to be explored: (1) down-regulation of T cell signaling pathways, connectivity among numerous ligands, and synergistic mechanism exploration with other immunoassays; (2) Whether LAG-3 can be combined with other therapeutic modalities, including chemotherapy, targeted therapy, and interventional therapy, to improve the effectiveness of tumor therapy; (3) Why LAG-3 is less effective than PD-1 under different signal transduction. Therefore, we need to use modern advanced biotechnology to optimize the molecular structure of LAG-3 inhibitors, clarify the functional and molecular mechanism characteristics of LAG-3 in more detail, and design more reasonable LAG-3 targeted therapy for various malignant tumors.
